# A special issue of *Essays in Biochemistry* on structural mass spectrometry

**DOI:** 10.1042/EBC20230006

**Published:** 2023-03-29

**Authors:** Hannah M. Britt, Rebecca Beveridge, Antonio N. Calabrese

**Affiliations:** 1 Physical and Theoretical Chemistry Laboratory, Department of Chemistry, University of Oxford, Oxford OX1 3QZ, U.K.; 2 The Kavli Institute for Nanoscience Discovery, Sherrington Road, Oxford OX1 3QU, U.K.; 3 Department of Pure and Applied Chemistry, University of Strathclyde, Glasgow G1 1XL, U.K.; 4 Astbury Centre for Structural Molecular Biology, School of Molecular and Cellular Biology, Faculty of Biological Sciences, University of Leeds, Leeds LS2 9JT, U.K.

**Keywords:** biochemical techniques and resources, mass spectrometry, structural biology

## Abstract

Mass spectrometry (MS) is now established as an analytical tool to interrogate the structure and dynamics of proteins and their assemblies. An array of MS-based technologies has been developed, with each providing unique information pertaining to protein structure, and forming the heart of integrative structural biology studies. This special issue includes a collection of review articles that discuss both established and emerging structural MS methodologies, along with examples of how these technologies are being deployed to interrogate protein structure and function. Combined, this collection highlights the immense potential of the structural MS toolkit in the study of molecular mechanisms underpinning cellular homeostasis and disease.

Structural mass spectrometry (MS) methods, also called structural proteomics methods, are being increasingly used to study the architecture of proteins and their assemblies [1–5]. An advantage of structural MS tools is that they can be deployed to study challenging systems that are intractable by other methods, including intrinsically disordered proteins, membrane proteins, complexes that exist in dynamic mixtures, and assemblies of proteins with nucleic acids and/or small molecules. Moreover, advancements have been made in applying structural MS methods in complex matrices, including cells, tissues, and lysates, and thus they are considered emerging tools for *in-situ* structural biology [6,7]. Structural MS methods can also be used in combination with other experimental approaches (e.g., cryoelectron microscopy, X-ray crystallography, and/or NMR spectroscopy) to generate complementary datasets that together can reveal mechanistic insights – so-called integrative structural biology [8].

A variety of MS-based tools to study protein structure are available, and whilst these methods are all linked by the instrumentation that provides the data read-out, i.e., a mass spectrometer, each has unique requirements for sample preparation, data acquisition, and data processing. Examples of structural MS methods include cross-linking (XL), hydrogen–deuterium exchange (HDX), native, ion mobility (IM), covalent labelling, and top-down MS. To better enable the newcomer to navigate this broad and exciting field we have, in this issue of *Essays in Biochemistry*, assembled a collection of review articles that summarise several of the key structural MS methodologies. Examples of applications of structural MS to address important biological questions are given, and cutting-edge methodological developments are described that will underpin our ability, in the future, to interrogate increasingly challenging phenomena.

Broadly speaking, structural MS methods are classified into one of two categories, based on whether they employ mass spectrometric analyses of intact proteins (top-down) or digested peptides (bottom-up). For intact protein analysis, proteins can be introduced into the mass spectrometer in either denatured or native states. Both denatured and native analyses can be used to reveal and interrogate proteoforms, including by identifying splice variants of proteins and co-occurring post-translational modifications, as highlighted by Habeck and Lermyte [9]. In native MS experiments, nondenaturing sample preparation (typically by reconstituting samples into aqueous ammonium acetate), and gentle instrument conditions during ionisation, are used so that aspects of protein tertiary and quaternary structure are retained in the mass spectrometer. In a native MS experiment, the stoichiometry of proteins and their assemblies can be studied, along with interactions with lipids and small molecules [10]. When combined with IM, insights into the architecture of proteins and their assemblies can also be gained [11]. This technology has found broad application, and in this issue, Oluwole et al. [12] highlight the advances in native MS methodologies that have enabled the study of challenging membrane proteins. Similarly, native MS has a theoretically unlimited size upper limit, and can be deployed to the study of very large assemblies. This includes the study of MDa viral particles using methods such as charge detection MS, as described by Miller and Jarrold [13]. While conventional native MS experiments require stringent buffer exchange, developments have also enabled the direct sampling of biomolecules from surfaces for both native MS and denaturing MS experiments, including tissue segments, as outlined by Wong et al. [14].

Another advantage of the mass spectrometer is that it can be used as a filter to select individual ion species for transmission/detection. As such, new research is now working towards combining native MS, including mass filtering of selected protein/assemblies, with imaging technologies. One example of this application is the combination of native MS with low-energy electron holography, as described here by Ochner et al. [15], to bridge the gap between MS and molecular structure determination.

HDX-MS is a bottom-up MS method that requires the protein-containing solution to be diluted with deuterium-containing buffer, resulting in the exchange of hydrogen atoms with deuterium from the solvent (only main chain amide hydrogens are detected in these experiments). The exchange reaction is allowed to proceed for a defined time before it is quenched and the protein digested. Deuterium incorporation is then measured by MS, which provides a readout of solvent accessibility and/or hydrogen bonding of specific regions of the protein sequence, and enables comparison of protein dynamics in different samples, for example, upon ligand binding. In this issue, Vinciauskaite and Masson [16] describe some of the key fundamentals that underlie HDX experiments, and Javed, Griffiths, and Politis [17] describe methodological advances that are being developed for the study of membrane proteins. Performing HDX reactions at timescales comparable to those of many biological processes is also challenging, (e.g., protein dynamics, enzyme reactions) (micro-msec) and advances in rapid HDX methodologies are now emerging to address this challenge, as described here by Chow et al. [18].

XL-MS is an additional bottom-up MS method that involves the addition of a suitable XL reagent with two reactive groups separated by a spacer arm to a sample, to covalently link proximal amino acid residues. The identity of these cross-linked residues can then determined by MS. The spacer arm of the reagent constitutes a distance restraint between these cross-linked residues that can be deployed to model structures, validate models, or compare with other data [19,20]. XL-MS technology has found broad application, including for the study of proteins/assemblies *in vitro* and in cell, but faces several analytical challenges as outlined here by Lee and O’Reilly [21]. Similarly, new advances are occurring to study protein–nucleic acid assemblies using XL-MS methodologies as described by Steinmetz et al. [22].

An advantage of structural MS is that the range of tools that have been developed means that many challenging biological questions can be addressed. As well as being individually informative, they can also be deployed during integrative structural studies, which harness the combined powers of several methods. Here, examples of structural MS applied to the challenging problems of protein aggregation [23], multidrug efflux systems [24], and cyanobacterial proteins [25] are outlined, to demonstrate the breadth of important questions that current applied research is tackling.

Combined, the range of methodologies and applications discussed in these review articles highlights the array of biological questions that can be addressed with structural MS, and some of the key considerations when designing structural MS experiments. We hope that you enjoy this special issue of *Essays in Biochemistry*, and that these reviews will stimulate new and exciting avenues of research where structural MS will underpin both discovery and translational research.

## Open Access

Open access for the present article was enabled by the participation of University of Leeds in an all-inclusive *Read & Publish* agreement with Portland Press and the Biochemical Society under a transformative agreement with JISC.

## Abbreviations

HDX: hydrogen–deuterium exchange
IM: ion mobility
MS: mass spectrometry
XL: cross-linking
